# Interpreting Mobile and Handheld Air Sensor Readings in Relation to Air Quality Standards and Health Effect Reference Values: Tackling the Challenges

**DOI:** 10.3390/atmos8100182

**Published:** 2017-09-21

**Authors:** George M. Woodall, Mark D. Hoover, Ronald Williams, Kristen Benedict, Martin Harper, Jhy-Charm Soo, Annie M. Jarabek, Michael J. Stewart, James S. Brown, Janis E. Hulla, Motria Caudill, Andrea L. Clements, Amanda Kaufman, Alison J. Parker, Martha Keating, David Balshaw, Kevin Garrahan, Laureen Burton, Sheila Batka, Vijay S. Limaye, Pertti J. Hakkinen, Bob Thompson

**Affiliations:** 1 Environmental Protection Agency, Research Triangle Park, NC 27711, USA; 2 National Institute for Occupational Safety and Health, Morgantown, WV 26505, USA; 3 Army Corps of Engineers, Sacramento, CA 95814, USA; 4 Agency for Toxic Substances and Disease Registry, Atlanta, GA 30329, USA; 5 ORISE Fellow hosted by U.S. Environmental Protection Agency, Washington, DC 20004, USA; 6 National Institute for Environmental Health Sciences, Research Triangle Park, NC 27709, USA; 7 Environmental Protection Agency, Washington, DC 20004, USA; 8 Environmental Protection Agency, Chicago, IL 60605, USA; 9 National Library of Medicine, Bethesda, MD 20892, USA

**Keywords:** air pollutants, ambient air, indoor air, citizen science, toxic chemicals

## Abstract

The US Environmental Protection Agency (EPA) and other federal agencies face a number of challenges in interpreting and reconciling short-duration (seconds to minutes) readings from mobile and handheld air sensors with the longer duration averages (hours to days) associated with the National Ambient Air Quality Standards (NAAQS) for the criteria pollutants-particulate matter (PM), ozone, carbon monoxide, lead, nitrogen oxides, and sulfur oxides. Similar issues are equally relevant to the hazardous air pollutants (HAPs) where chemical-specific health effect reference values are the best indicators of exposure limits; values which are often based on a lifetime of continuous exposure. A multi-agency, staff-level Air Sensors Health Group (ASHG) was convened in 2013. ASHG represents a multi-institutional collaboration of Federal agencies devoted to discovery and discussion of sensor technologies, interpretation of sensor data, defining the state of sensor-related science across each institution, and provides consultation on how sensors might effectively be used to meet a wide range of research and decision support needs. ASHG focuses on several fronts: improving the understanding of what hand-held sensor technologies may be able to deliver; communicating what hand-held sensor readings can provide to a number of audiences; the challenges of how to integrate data generated by multiple entities using new and unproven technologies; and defining best practices in communicating health-related messages to various audiences. This review summarizes the challenges, successes, and promising tools of those initial ASHG efforts and Federal agency progress on crafting similar products for use with other NAAQS pollutants and the HAPs. NOTE: The opinions expressed are those of the authors and do not necessary represent the opinions of their Federal Agencies or the US Government. Mention of product names does not constitute endorsement.

## 1. Introduction

The *Air Sensors 2013: Data Quality and Applications* workshop, held in Research Triangle Park, N.C., highlighted the substantial advances in the development of portable air sensors capable of providing real-time measurements of ambient air pollution [1]. One anticipated benefit for the use of air sensors is the potential to expand upon the already well-established network of air quality monitors for key air pollutants. Sensor manufacturers introduced a number of new, relatively low-cost, portable air sensors capable of continuously measuring ambient levels of ozone (O_3_) and nitrogen dioxide (NO_2_). In addition, considerable progress was described regarding the development of air sensors capable of measuring ambient concentrations of particulate matter (PM), and total volatile organic compounds (VOCs). Since that 2013 workshop, the field of portable air quality sensing technology has continued to evolve at a rapid pace, with commercially available products now available for O_3_, NO_2_, PM, VOCs, as well as for other pollutants, both alone and in complex mixtures. While the potential for these technologies continues to be great, significant challenges in their application remain. Most notably, there is a wide range of data quality differences between different sensor manufactures and models [2], and there is a significant question about how air quality data collected on timescales as short as 1-min should be interpreted in comparison to the available health reference values for air pollutants which are typically based on exposure durations of several hours to many years [3]. For example, commercially available portable air sensors for O_3_ can provide the user with minute-by-minute O_3_ concentrations; however, interpreting these very short-term measurements with respect to potential adverse health effects is difficult. The health-based National Ambient Air Quality Standard (NAAQS) for O_3_ is based on an 8-h average concentration which is backed by thousands of studies from numerous independent researchers and is established through a rigorous process to be scientifically defensible and legally enforceable; no such standards have been developed for these shorter duration exposures. Similar challenges exist when applying Occupational Safety and Health Administration (OSHA) standards. The most comparable OSHA exposure standards include the Permissible Exposure Limit (PEL), which is an 8-h time-weighted average, and the Short Term Exposure Limit (STEL), which is a 15-min time-weighted average.

Detection and monitoring of most non-NAAQS environmental chemicals (including the hazardous air pollutants), toxins and pathogens still largely involves identifying each individual agent, which often requires sending samples to a remote analytical laboratory for analyses. Delivery of laboratory results may take days, weeks or even months. Although the deployment of portable direct-reading instruments, such as photon ionization detectors (PIDs) for total VOCs, can provide some real-time information, these screening instruments lack the sensitivity or selectivity delivered by analytical laboratories and thus, are unable to fully inform a user with a critical need for high-specificity (e.g., rapid response decision-makers). New technologies such as miniaturized light emitting diodes, ultra-violet light detectors and functionalized graphene resistors have enabled the development of chemical detectors capable of delivering laboratory-quality analyses in near real-time. Coupling these types of sensors with global positioning and cell phone technologies may enable the detection, quantification, and visual monitoring of environmental contamination in real-time from remote locations. These promising technologies are expensive, however, and will likely remain out of reach for all but the most dedicated citizen scientist. As described in the following sections, understanding and advancing these types of applications has been a focus of the ASHG: beginning with defining the relevance of air sensors; facing the challenges of communicating across diverse key audiences; dealing with data validity issues; and moving toward a future with more reliable and useful sensor readings.

### Formation of the Air Sensors Health Group (ASHG)

Recognizing the potential widespread use of portable air sensors, the likely data interpretation challenges these sensors would present to state and local governments, and the opportunity for collaboration across Federal agencies, the ASHG was formed in 2013. The ASHG is a multi-institutional collaboration of Federal agencies devoted to keeping abreast of new sensor technologies and to assist in the proper interpretation of sensor data as potential indicators of air quality. ASHG consists of experts in a number of areas, including toxicology, public health, engineering, monitoring and sampling, ambient air, indoor air, and occupational health, to name a few.

The ASHG monitors the state of the science at each institution and aims to find common ground on how sensors might effectively be used to meet a wide range of research needs in the occupational, indoor air, and ambient air settings. The ASHG also aims to be a resource for state, regional, and tribal organizations, as well as for citizen scientists and community members considering the use of portable air sensors for air pollution research and decision-making. To date, the ASHG has focused on multiple fronts: assisting EPA Program Offices in developing tools and message statements regarding the potential for adverse health effects from the short-duration air sensor readings for PM and O_3_; providing analysis comparing short-term readings to longer-duration averages from existing official monitoring stations used in determining compliance to the NAAQS; and developing prototype visual tools to assist in communicating appropriate interpretive messages. These ASHG contributions have been incorporated into projects and programmatic products which are discussed in later sections of this paper. This review summarizes the challenges, and successes of those initial ASHG efforts, and Federal agency progress on crafting similar products for use with other pollutants.

## 2. Challenges

### 2.1. Relevance of Sensor Measurements

A critical concern for interpreting readings from air quality sensors is to have a realistic understanding of what the sensors are actually measuring; how that relates to the expectations of users (including citizen scientists, researchers, regulatory agencies, etc.) for sensitivity, specificity, and robustness of the intended application; and the extent to which those measurements of exposure can appropriately be used to communicate potential hazard and manage risks to public health. The magnitude of this concern varies greatly across the types of pollutants purportedly being measured and is related to a number of factors, such as the chemical and physical nature of the pollutant, the reactivity of the pollutant, concentration and compositional changes over time and location, and the influences with various atmospheric conditions. EPA guidance on achieving high-quality data through systematic planning using the data quality objectives process can be found online [4]. Standards for data quality can differ slightly depending on the context; additional standards are discussed as those contexts are examined in the ensuing sections of this review. The ASHG recognized early in their discussions that this ideal for collecting high-quality data may not be readily obtainable for all potential users of air pollutant sensors.

Low-cost air sensors on the market to date are predominantly of three types; optical, electrochemical, or metal oxide. Generally, these sensor types are not as specific and do not incorporate the front end conditioning or selection that is present in Federal Reference Method (FRM) or Federal Equivalent Method (FEM) measurement devices. Therefore, low-cost sensor measurements may suffer from the influence of co-responsive pollutants, environmental conditions, and even sensor component production variations. The need to understand what the sensor is measuring is of prime importance. In order to better understand the measurement and potential confounding influences, an informed user must understand the physical and chemical nature of a pollutant of interest and how it responds to the environment as well as the sensor measurement technology itself. Field testing may help a user understand the influence of confounding factors and could identify co-responsive pollutants. The interpretation and use of low-cost air sensor data will be enhanced by simultaneous collection of co-responsive pollutant concentrations and environmental data including temperature, relative humidity, wind, and weather (i.e., precipitation, fog), as well as observations about local pollution sources. In this context, chemical sensors must be validated for chemical specificity and sensitivity under the environmental conditions expected in the field prior to any consideration for demonstrating regulatory compliance.

#### 2.1.1. Interpreting Sensor Readings

Managing expectations about what sensor measurement data can and cannot be used for is paramount in communicating with both citizen scientists and the general public at large, who for a variety of reasons (e.g., personal health, general air quality interest) may, in the future, routinely consult a sensor. In anticipation of this interest on the part of the public, the U.S. EPA began a pilot effort in June 2016, to begin addressing the interpretation of short-term sensor readings in the context of air quality [5]. There are several challenges to interpreting these data. Among these challenges are sensor performance and the short-term, sometimes instantaneous output from a sensor. Short-term sensor data come from instruments of unknown performance quality and, importantly, these short-term concentrations cannot be compared to the NAAQS to draw conclusions about what these nearly instantaneous exposures may mean in terms of health impacts. The NAAQS are based on longer exposure durations (e.g., 8-h or 24-h averages) consistent with the health evidence from the reviews of these standards. This health evidence does not support linking 1-min (or shorter) ozone or PM_2.5_ concentrations to adverse health effects, thus a 1-min sensor reading is not directly comparable to the NAAQS, or to the related Air Quality Index (AQI) categories.

To help the public understand the implications of these readings, scientists at EPA have piloted a color-coded Sensor Scale that might be used in conjunction with the AQI to help the public better understand what their short-term sensor data mean in the context of local and regional air quality and consequently make behavioral decisions about outdoor activities. Statistical approaches were used to understand the relationship between short-term ozone and fine particulate matter measurements with longer term averages. The Sensor Scales and explanatory background materials are housed in the Air Sensor Toolbox on the EPA website [6] and have also been described elsewhere. To test the effectiveness of these messages and their visual presentation, EPA conducted focus groups in 2015 and 2017, before and after the deployment of the pilot respectively. Analysis of the outcomes of these focus groups is underway. After considering the input from the focus groups, EPA will refine the messages as appropriate and consider outreach to air quality sensor developers on these focus group findings. Additional work is also on-going to develop similar message schemes for selected HAPs.

In addition to the NAAQS, EPA’s Integrated Risk Information System (IRIS) provides inhalation reference concentration (RfC) values for HAPs and other key pollutants important to many EPA Programs with a focus on chronic exposure durations (from years to a lifetime). The Agency for Toxic Substances and Disease Registry (ATSDR), a federal public health agency of the U.S. Department of Health and Human Services, develops similar substance-specific minimal risk level (MRL) values for acute (1–14 days), intermediate (15–364 days), and chronic (365 days and longer) exposure durations [7]. ATSDR’s work is focused on Superfund sites, but environmental health specialists apply the MRLs in a wide range of investigations. Many state agencies also develop inhalation health effect reference values in support of their programmatic needs. More generically, the term reference value is used in this text to include all of the various values referred to as standards, guideline values, toxicity values, health benchmarks, etc., and includes values developed for use in emergency response, occupational exposure monitoring, and those protective of the general public. Additional reports are available for a more complete comparison of the available systems of health effect reference values [3,8], and between values for specific pollutants [3,9–13].

#### 2.1.2. Occupational Versus Environmental Exposures

One issue likely to be resolved on a case-by-case basis is the overlap between the environment and the workplace. An employee may be exposed to a chemical in the workplace at a higher concentration than in an outdoor environment. In addition, because the worker is assumed to be fit and healthy and to have periods of recovery from exposure, the levels of concentration deemed tolerable are also higher. A citizen wearing a sensor outdoors, may well carry it into their workplace, and could then experience problems reconciling the levels of pollutants considered acceptable in these different milieus. For example, a sensor designed to help citizens avoid pollution from motor vehicles might measure carbon monoxide. EPA sets a primary NAAQS of 9 ppm averaged over an 8-h period and 35 ppm averaged over a 1-h period, not to be exceeded more than once per year. In the workplace, the situation is not quite as straightforward, but all the limit values which might be applied are higher than those under the NAAQS. The OSHA Permissible Exposure Limit is 50 ppm, while the National Institute for Occupational Safety and Health (NIOSH) Recommended Exposure Limit is 35 ppm, and the American Conference of Governmental Hygienists (ACGIH) Threshold Limit Value is 25 ppm, all averaged over an 8-h period, as with the 9 ppm NAAQS standard. If the monitor alarm is set for the 8-h average NAAQS value, or possibly even at the one-hour average value, the alarm might easily be triggered in the workplace, which might be, for example, a bus garage or foundry. The ensuing discussion with the workplace safety manager or the employer regarding the acceptability of the exposure situation might be difficult for both parties in the absence of well-thought out responses. Nevertheless, in a holistic vision of the exposome, which should consider all exposures over all life-stages, it may not be appropriate to regard ambient, indoor and workplace exposures as somehow “different”, to be always measured and assessed separately [14], and so it is to be hoped that it will not be necessary to turn off ambient continuous air monitors “at the factory gates”.

#### 2.1.3. Global/International Perspectives

Low cost air quality sensors are of interest worldwide. Multiple studies have distributed sensors in an effort to apply themto local environmental research [15–18]. One of themost notable efforts involving multiple countries and metropolitan areas was the Citi-Sense project [19,20]. This diverse research project involved technology developers, citizen scientists, academics, and professional research organizations using low cost sensor technologies with a purposeful intent [21,22]. The pan-European initiative, EuNetAir, has goals of developing harmony in sensor selection and deployment strategies as well as coordinating sensor evaluation protocols to ensure the timely integration of air quality sensors into monitoring networks [23–25]. The Clean Air Asia consortium, a partnership of multiple Asian-based cities, sensor enthusiasts, academics, and air quality professionals are attempting to improve air quality and improve the overall living conditions in some of the most polluted cities in the world [26].

Even with the apparent world-wide enthusiastic use of low cost sensors to inform public awareness of environmental conditions, there is also a call to ensure the data being collected from these devices across the globe are accurate enough to be used in a purposeful manner [27]. This call for an adequate understanding of sensor performance is not only a reasonable approach but one that must be pursued with the same enthusiasm as those wishing to disseminate low cost sensors to a global population.

A multitude of pseudo air quality index messaging applications are available on the internet from sources around the world [28]. Some of these applications are using low cost sensors to collect air quality data, potentially not accounting for the accuracy of the measurement or the environmental setting in which the measurements are taking place (e.g., indoor, outdoor, near source categories). Furthermore, use of subjective data messaging on health impacts of such sensor measurements without a scientific basis has the potential of confusing the public-at-large and their understanding of sound air quality awareness indices.

The World Health Organization (WHO) describes air pollution as a “major environmental risk to health”. The WHO Air Quality Guidelines (AQG) provide an assessment of air pollution health effects and recommend reference values for health-harmful pollution levels of ozone, PM_10_, PM_2.5_, SO_2_, and NO_2_ [29]. ATSDR has considered AQGs in the screening process of health evaluations for multiple site investigations in recent years [30–33]. The European Commission (EC) has developed air quality standards for the six US criteria pollutants, as well as benzene, polycyclic aromatic hydrocarbons (PAHs), and three metals: arsenic, cadmium, and nickel.

### 2.2. Communicating Across Audiences

Portable sensors present a great deal of promise for identifying personal exposure to toxicants present in the environment. However, communication between government or public health agencies, sensor manufacturers, researchers, employers and employees, and citizen scientists is particularly important given the varying degrees of accuracy across sensors, the complexities of environmental exposure, and the difficulties in interpreting potential health risk based on readings from a portable sensor. Thus, the subsections below summarize the various efforts that agencies represented on the ASHG membership have put toward communicating with these groups.

#### 2.2.1. Citizen Scientists and Communities

Over the last decade, members of the public have become increasingly engaged in taking measurements of their environments and otherwise contributing to scientific research. Enabled by the rapid pace of growth of sensor technology, many citizen scientists collect data on air quality in their local environments, both individually and as a part of organized projects. The growth of these technologies and a surge in enthusiasm for these approaches has pushed the boundaries of traditional institution-driven research. Often called “citizen science”, these efforts are also referred to as civic or community science, community-based monitoring, crowdmapping, participatory science, open science, or crowdsourcing. In *citizen science*, the public participates voluntarily in the scientific process, addressing problems in ways that may include formulating research questions, conducting scientific experiments, collecting and analyzing data, interpreting results, making new discoveries, developing technologies and applications, and solving complex problems [34]. Of particular relevance to air quality research and air sensors, *community science or community citizen science* is “collaboratively led scientific investigation and exploration to address community-defined questions, allowing for engagement in the entirety of the scientific process. Unique in comparison to citizen science, community science may or may not include partnerships with professional scientists, emphasizes the community’s ownership of research and access to resulting data, and orients toward community goals and working together in scalable networks to encourage collaborative learning and civic engagement” [35].

The growth of citizen science offers significant opportunities and challenges for federal agencies. Citizen science increases public understanding and community and civic engagement with science and environmental issues, especially locally. Citizen science can connect agencies to the public, provide opportunities for working together towards common goals, support innovation, and make science more accessible and available. It provides data that would otherwise be inaccessible, helps generate a more comprehensive understanding of variation over space and time, and can increase our understanding of social science and human behavior. At the same time, low cost sensor technologies and citizen science introduce challenges to federal agencies, such as communicating risk to project participants, increased public pressure for actions like regulations and enforcement, and communication of data quality. In December 2016, the National Advisory Council for Environmental Policy and Technology provided EPA with advice and recommendations for how to maximize the benefits of citizen science and respond to the corresponding challenges. Recommendations include building technical capacity, providing guidance and communicating data quality needs for different data uses, and integrating citizen science into the full range of EPA’s work [34].

EPA is collaborating with citizen scientists to share tools and technology, conduct air monitoring studies, and interpret sensor data. The Air Sensor Toolbox website [6] was created in 2014 to provide resources and tools to citizens interested in learning more about conducting successful air monitoring projects. Topics include how to use sensors, interpreting sensor data, information about EPA air monitoring projects, funding resources, and local air monitoring examples across the United States. The Air Sensor Guidebook [36] provides a comprehensive overview of air pollutants, sensors, study design, data collection, and data interpretation. Other resources include sensor evaluation reports highlighting performance of various sensors on the market, standard operating procedures for sensors, fact sheets, blogs, and training videos from the EPA-sponsored Community Air Monitoring Training in 2015.

Another way EPA engages communities is through collaborations. One such collaboration was conducted in the Ironbound community in Newark, New Jersey through a Regional Applied Research Effort (RARE) grant [37]. EPA scientists trained citizen scientists from the Ironbound Community Corporation (ICC) (The Ironbound section of Newark is a multicultural, multiracial mosaic whose population of 50,000 reflects the diversity and the challenges in urban America. ICC impacts the lives of nearly 1000 people daily and thousands annually. The majority of ICC’s 3000 annual clients are from very low to low income households with low literacy and English proficiency and multiple family stressors.) to operate sensor pods developed by the Office of Research and Development (ORD). The collaboration allowed for joint decisions regarding study design, sensor siting, and collection, validation and interpretation of sensor readings. Following the Ironbound project, a similar RARE grant collaborative project was performed in Ponce, Puerto Rico. EPA scientists used lessons learned from the first project to attempt to improve study design topics such as roles and responsibilities and data validation and interpretation. Tasks such as managing, validating, and interpreting large datasets can be an obstacle for community groups involved in air monitoring projects.

ATSDR and NIOSH have partnered with University of Cincinnati, Georgia Tech, and University of Texas Arlington to pilot the use of real-time sensors as sentinels that trigger sample collection via more conventional devices. For example, a low-budget hydrogen sulfide detector can be programmed such that, once a certain threshold is exceeded, a VOC canister will be filled and then sent for laboratory analysis. ATSDR is developing these projects either to characterize peak exposures or as the first stage in assessing the need for a full-blown traditional air monitoring study.

Sensor collocation is an important step to perform before embarking on any monitoring project, regardless of who is conducting the monitoring. This process involves siting sensors in line with regulatory monitors (the gold standard) for a period of time in order to compare the two datasets and establish a regression equation to normalize sensor data and make it more accurate. EPA scientists recently created tools designed to assist citizens in this process, including an easy-to-use Excel™macro that allows one to compare two datasets, such as sensor and reference data, and with one click provides a regression equation, comparative data graphs, and descriptive statistics. A training document that explains collocation and why it is important, and how to perform a proper collocation, accompanies the macro. EPA is partnering with the Clean Air Carolina community group and the Eastern Band of Cherokee Indians to conduct their own collocation projects, pilot test these resources and provide input on how to improve them for use by the general public.

#### 2.2.2. State, Local and Tribal Agencies

State, tribal and local health and regulatory agencies were identified as a primary target audience early in discussions within the ASHG. The more proximately located agencies and offices are more likely to be called by the public and press, and may be more resource-challenged to interpret data generated using sensors. The EPA Regional Offices were identified to be the most likely first contact point for EPA. Similarly, ATSDR has Regional Offices that are another resource for health agencies.

Health agencies at all levels of government are called upon by the public and press to interpret air monitoring data. City and county health departments, depending on their size and community needs, may have environmental health specialists on staff who are familiar with air monitoring methods. Frequently, local agencies will defer to their respective state health department, many of which have specialists that are supported by the ATSDR Cooperative Agreement Program. State or local agencies may in turn also contact ATSDR or its sister agency, the National Center for Environmental Health (NCEH), which is part of the Centers for Disease Control and Prevention (CDC). As noted earlier in Section 2.1.1, there are several reference value systems which have been applied in interpreting exposures to non-criteria air pollutants, including the RfC and MRL values. Health agencies also frequently make site-specific exposure dose calculations and may evaluate health risks for shorter averaging periods by adapting the dose-response information in toxicology studies on which the reference values are based.

In order to address these needs, EPA Regional Offices have initiated outreach to state, tribal, and local environmental agencies to facilitate information sharing amongst stakeholders and to develop an inventory of sensor-based community air quality investigations. EPA Regional Office staff have highlighted EPA’s Air Sensor Toolbox for Citizen Scientists [38] in this outreach, facilitating exchanges on sensor operation and maintenance, funding opportunities, and tools for data interpretation. For example, the Minnesota Pollution Control Agency provided input to the EPA Regional Office in Chicago (Region 5) on its own experience managing an air sensor loan program, and shared documentation it had developed to inform community members about proper sensor operation.

Monitoring for the NAAQS pollutants is delegated to state agencies with oversight from EPA Regional Offices and EPA’s Office of Air Quality Planning and Standards (OAQPS); there is a structured regulatory program to determine compliance status with each NAAQS. Emissions of HAP are regulated at the source of the emissions, and monitoring is mostly initiated at the local level (i.e., state, tribal and local agencies), with federal guidance and some federal funding. There are very specific regulatory requirements in monitoring for the NAAQS pollutants but the States and Tribes have more leeway in monitoring for other purposes (e.g., for HAPs). EPA’s Superfund Program uses various technologies for emergency response, and new sensors may be useful to inform decisions in these scenarios. Federally recognized Tribes interface with EPA and ATSDR on a government-to-government basis; many receive EPA grants or ATSDR technical assistance to do monitoring and other environmental health projects.

#### 2.2.3. Sensor Manufacturers

Low cost air quality sensors have exploded onto the commercial market in the last decade. Development and manufacturing of these devices is not isolated to a single sector or industry. Some of the earliest developers were design teams associated with academic industrial/design arts programs, telecommunication research groups, and non-profit citizen scientists [39]. In particular, these developers took advantage of inexpensive sensor components and their individual areas of expertise to craft air quality sensors to meet a wide variety of needs [40]. More traditional instrumentation manufacturers have recently started to invest research capital into the low cost sensor area [41]. Their presence is the result of the growing citizen science movement and an obvious market niche.

The existence of a multitude of sensor manufacturers has resulted in both obvious benefits and deficits. On the plus side, there are numerous low cost sensors in a price range typically well under $1000 available for the interested user for both particle as well as gas phase pollutant monitoring. Many offer user friendly features such as immediate data visualization through smart phone applications with wireless data transmission. Some concerns associated with low cost sensor production is that often manufacturers may lack the technical know-how or capabilities to adequately test or calibrate their devices prior to releasing them to the market [27]. Other concerns include reliability issues between replicate copies of the same sensors attributed to either a poor overall manufacturing process associated with the fully assembled sensor or issues with the individual components themselves [42,43].

Recent sensor evaluation efforts by recognized institutions, including the U.S. EPA and others [6,44] would appear to be having a positive impact upon sensor manufacturing. In particular, work conducted in 2012 resulted in some of the first reported performance tests of sensors from a wide variety of manufacturing sources [2]. The evaluations resulted in an almost immediate update of the technology by many of the manufacturers to overcome issues first revealed in the tests (e.g., battery failures, low detection sensitivities, poor telecommunications protocols). Some of those early manufacturers would appear to have left the market while other new ones have joined. These observations suggest that it is likely that more mature instrument manufacturers will continue to invest in this area and capture more of the market share as start-up groups continue to develop unique devices to meet a particular niche.

### 2.3. Calibration and Validation

As mentioned previously, the calibration procedures conducted by most low-cost sensor manufacturers are often severely inadequate or non-existent and fail to establish the sensor’s performance characteristics, especially when used in the ambient environment. End users and research organizations have often taken the lead on establishing the performance of these emerging technologies and sharing their results. Noted groups establishing sensor performance include the European Union’s Joint Research Center [45], the U.S. EPA [46], and the South Coast Air Quality Management District’s AQ-SPEC laboratory [47]. Results show that while nephelometric (light scattering) devices might perform well in direct chamber-based evaluations, they often reveal significant departures from a true reference grade instrument response under real-world (ambient) conditions [1]. In like manner, while excellent chamber-based response relationships have been observed for select gas phase pollutants [2], ambient test results have been less promising or inconsistent [42]. There are of course, exceptions to these observations and certain devices and sensors appear to show more promise relative to their performance features [48].

Gas detection in the workplace has a long history of being driven by flammability concerns, beginning with the miners’ safety lamp, but direct-reading real-time instruments have also been used to determine toxic gases for almost 100 years; colorimetric detector tubes patented for detecting carbon monoxide in the 1930’s are examples. More recent developments of infra-red analyzers and gas chromatography detectors (with or without an associated gas chromatograph) have been portable, but not personal, although current research into miniaturization may alter that situation. Electrochemical cells have been developed for specific acute hazards, such as carbon monoxide, hydrogen sulfide and chlorine. These are used in process safety applications, but have also been adapted for use in personal dosimeters. The quality of data used for safety monitoring and personal exposure assessment in the workplace is of paramount importance, particularly in relation to acutely hazardous substances. The European Union countries have developed guidance for the performance, testing, selection, installation, use and maintenance of electrical apparatus used for the direct detection and direct concentration of toxic gases and vapors. Other Standards-setting organizations have published similar products, and a joint Working group of the International Organization for Standardization (ISO) and the International Electrotechnical Commission (IEC) are working on umbrella standards to replace these (these Standards are detailed in Appendix A). In the USA, NIOSH has developed guidance for ensuring data quality for gas and vapor detection, including during emergency response [49,50]. The American Industrial Hygiene Association, through their Gas and Vapor Detection Systems Committee (now Real-Time Detection Systems Committee) has developed guidance for manufactures to report specifications for electronic real-time gas and vapor detection equipment [51].

Recognizing the need to calibrate instrument response under real-world conditions, variable performance based on changing conditions (e.g., aerosol size-distribution), and deteriorating sensor response as due to age, new research efforts have been focused on the idea of auto-calibration methods [52]. Such methods may be able to maintain the accuracy of a node-based distributed sensor system for longer term measurements.

Extending beyond the need for validation in controlled laboratory settings to “real world” conditions and environmental epidemiological and citizen science applications, the National Institute for Environmental Health Sciences (NIEHS) released a phased field validation program in 2013 [53]. Through this effort, teams of engineers, exposure scientists and environmental epidemiologists are working together to test sensor performance at the pilot scale, including iterative improvements in sensor system design and performance and then scaling up the application to a full-sized epidemiological study to demonstrate the added value of temporally and spatially resolved air pollution exposure assessment relative to existing ‘citizen’ measures.

## 3. Related Projects and Programs

Although the ASHG has not been a prime mover in development of key products or programs, it has served as an advisory group and has contributed to a number of projects and programs, as noted in the sections below.

### 3.1. Village Green

The U.S. EPA’s Village Green project has provided a test-bed for long-term evaluation and application of emerging air quality sensors in a variety of community settings [54]. While the sensor technologies for particulate matter and ozone are not considered truly low cost (~$6000/each), they do represent mid-tier technologies [55] which are showing good to excellent capabilities for certain attributes. In particular, they are capable of providing extended periods (months) of ambient air quality monitoring using sustainable energy (solar power) with little or no technical support and often with a high degree of agreement with local reference monitoring [56]. Another unique feature of the Village Green is that it was designed to stream data continuously to the public via its web-based data portal. It was in fact, the first real-time public reporting of air quality data for environmental awareness purposes provided to the general public by the US EPA. Since its conception in 2014, Village Green stations have now been deployed in a total of eight (8) U.S. metropolitan areas and involve a variety of air quality sensor and emerging technologies (variable sustainable power supplies, data microprocessing features, etc.).

At the time of its development as a technology test bed and community air quality awareness tool, its capabilities to meet purposeful air quality data analyses were not established. EPA’s investigations into short time interval sensor data messaging provided an opportunity to use the Village Green for such an analysis due to its extensive database containing 1-min measurements of both ozone and fine particulate matter. Analysis of that database resulted in a pilot Sensor Scale associated with potential short time interval air quality measurements [5]. EPA is launching a pilot project to test a new tool for making instantaneous outdoor air quality data useful for the public. The new “Sensor Scale” is designed to be used with air quality sensors that provide data in short time increments–often as little as one minute [5]. EPA developed the scale to help people understand the 1-min data the stations provide and how to use those data as an additional tool for planning outdoor activities.

### 3.2. The E-Enterprise Advanced Monitoring Team (EEAMT)

Under the direction of the E-Enterprise Leadership Council, a joint EPA/State Advanced Monitoring team was formed in April 2015 to address the challenges and opportunities presented by rapidly changing air and water technologies. Data interpretation is one of five priority projects identified in the path forward for EPA, States, and Tribes [57]. In order to provide context and interpretation of advanced monitoring data in formats relevant and understandable to users, the team was charged with advancing (1) statistical analyses to understand the relationship between continuous data and data collected over longer-term averaging times or via discrete (e.g., bi-weekly) sampling; (2) development of visualization tools (e.g., interactive maps) and websites with appropriate messaging; and (3) development of outreach and communication materials.

Conducting statistical analyses to understand the relationship between short term measurements and longer term standards or discrete measurements is a blanket need across media and pollutants. While messaging already exists to alert the public about air or water quality conditions experienced over specific time periods (e.g., 1-h, 8-h, or 24-h), the same messaging should not be used to translate short term (e.g., 1-min) measurements. Two articles discussing an approach to relate short term ozone and particulate matter (PM_2.5_) measurements with longer term averages have been published [58,59]. Other needs identified by the team include data analysis and messaging for SO_2_, NO_2_, CO, PM_10_, benzene, total volatile organic compounds (VOCs), and other specific VOC compounds.

In order to create visualization tools that make continuous and discrete state/federal data more accessible and understandable, EPA and States have determined it is important to present real-time (e.g., hourly concentration values) along with appropriate caveats. Examples of caveats include marking data as “raw”, “provisional”, or “final” and distinguishing between different forms of data (e.g., regulatory vs. peer reviewed). A need exists to display continuous data along with discrete measurements while giving the data context including geographical and meteorological information. Mockups were drafted as part of a thought process to address these issues and allow for EPA and States to provide guidance or expertise to big data developers desiring to stream, collect, or use regulatory and sensor data. Once short term messaging has been developed, it is important to develop appropriate outreach and communication materials that EPA and States can use for consistent messaging including frequently asked questions (FAQs) and a standardized, centralized repository of metrics that break down toxicity/health information by pollutant for both short term and long term effects (e.g., gaseous, metals, PM, pH, toxins, dissolved oxygen, etc.). Outreach material should describe the limitations of sensors and sensor networks including information on what we do not know and cannot measure with confidence.

### 3.3. Homeland Security Applications

Air sensors have many important applications relating to homeland security, but two roles are especially important: (1) serving as sentinels to detect the release of dangerous substances into the air, and (2) providing measurements of the amount of dangerous substances present in the air so that health risks can then be assessed and appropriate decisions made to protect public health.

Serving as sentinels to provide early warning of the release of dangerous substances is a very critical function for real-time air sensors. In this capacity, air sensors are designed to monitor background levels of various substances (dangerous chemicals or/or their indicators) and detect any significant increases in their presence. Air sensor networks have already been deployed extensively as sentinels in several major cities, chemical plants, military installations, and during major public events. These networks have been deployed on roof tops, in underground transportation systems, and in various types of mobile ground vehicles and aircraft.

In addition to their role as sentinels to detect the release of dangerous chemicals, air sensors may be used to measure concentrations of harmful chemicals which can potentially provide an estimate of health risks to persons breathing the air. Estimating health risks from acute or short-term exposure durations is a challenging task, as has already been noted in this paper. Most of the tools used to assess health risks are more centered on long-term, chronic, or lifetime exposure to chemicals. For example, as EPA’s premier program for assessing health risks from chemicals, the Integrated Risk Information System (IRIS) has focused on dose-response data for chronic exposures. Health effect reference values derived to assess long-term health risks have very little, if any, value for assessing health risks from acute, short-term, or intermittent exposure scenarios, and few have been developed to assess those health risks to the general public.

A number of chemical release accidents, including the December 1984 accidental release of methyl isocyanate from a Union Carbide chemical plant in Bhopal, India, led to the formation of the Acute Exposure Guideline Level (AEGL) program in 1995 [60]. Standing Operating Procedures for deriving AEGL values were developed by the National Research Council (NRC) [61] for use in both planning for and during a catastrophic chemical release event, using values covering inhalation exposures from 10-min to 8-h.

Shortly after the events of 11 September 2001, the need for additional sets of values for use for remediation in the aftermath of such events led to the development of another set of health metrics, called Provisional Advisory Levels (PALs). PALs were derived by building upon the AEGL methodology developed by the NRC [61], to create values for use at 1-day, 30-days, 90-days, and 2-years durations [62]. Under the PALs program, EPA has drafted over 3000 numeric values for over one hundred priority chemical agents, two routes of exposure (ingestion and inhalation), three levels of harmful effects, and four relatively short exposure durations. These values, in addition to those already published by many other organizations [3], can provide a context on which to compare sensor-derived exposure levels.

### 3.4. Indoor Air–Non-Industrial

Indoor air quality (IAQ), which is the air quality within buildings (i.e., homes, schools, offices and other non-industrial buildings) and other enclosed spaces, can affect the health, comfort and ability to perform for occupants, from infants to senior citizens. IAQ involves many factors including: outdoor air contributions; chemical, micro-biological, and particulate contaminants; and, characteristics of the indoor climate such as temperature, humidity and airflow. Americans spend about 90 percent of their time indoors [63–66], where pollutant levels, like some VOCs, may be two to five times higher—and occasionally 100 times higher than outdoors [67–69]. EPA traditionally has focused its IAQ efforts to identify sources and developing guidance to reduce human exposure to unhealthy indoor air and to provide low-cost mitigation strategies consistent with public health practices. In addition, many international professional, trade, and standards organizations have dedicated committees addressing a broad range of indoor air quality issues. With the increased availability of low-cost sensor technology, however, the resources we use to assess indoor air have been expanding.

One challenge for sensor work within indoor environments is the fact that the several pollutants important in the indoor environment cannot be detected accurately by current low-cost sensor technology. Indoor air sensors have the same challenges that ambient air sensors encounter when attempting to evaluate short-term sensor readings. Most of the indoor air pollutants also share the challenge associated with HAPs, the lack of enforceable or agreed upon health based standards to with which to compare sensor readings. As with HAPs, the available health effect reference values may be inappropriate for direct use with indoor air sensor readings. There are several other challenges unique to using sensors indoors—for example, unlike the ability of ambient air sensors to be collocated with regulatory monitors to help assess accuracy, there is no similar availability for indoor air.

While guidance development around IAQ best practices will continue within EPA, the potential for increased use of sensor technology for indoor air quality continues to evolve. Sensors that work with occupants’ health-assessment technologies and those that can actuate building systems or individual appliances automatically may help enhance IAQ efforts in the built environment. There is a great potential that, as low-cost sensor technology improves, it may be a viable complement to comprehensive building system approaches currently used by the IAQ community and would help inform and improve IAQ stakeholders’ ability (both professional and consumer level) to use IAQ management tools, interpret air quality data for their space, and assess the benefits of IAQ-related actions.

It is important that we not only continue the engineering “fixes” that are constantly improving source control, as well as ventilation and filtration/air cleaning systems, but also work to improve sensor technology as a tool to help optimize building performance and increase the accessibility of these tools as a complement to integrated strategies for improving IAQ for all parts of society. Within the context of IAQ, this may involve creating future systems that can be automated, improving the integration of these low-cost tools with building and health-assessment systems, establishing guidance that creates IAQ best practices that integrates these tools into existing public health practices and provides appropriate messaging for the public to better understand their health in their indoor environments.

## 4. Maximizing the Usefulness of Sensor Readings

As discussions within the ASHG have progressed over the years, approaches have been considered on how sensors may improve our understanding of real-world dosimetry, and how we might better organize the process of developing and using sensors through life-cycle analysis. Both of these approaches are more immediately relevant to research-grade sensor technologies; however, as more improvements in sensitivity and specificity are made to the low-cost sensors available to citizen scientists, these approaches will apply to a more universal set of sensor users.

### 4.1. Transitioning Sensors into Dosimeters and Future of Exposure Science

Radiation biology provides a paradigm for transitioning sensor-derived data into estimates of internal dose. Biological effect data from early animal studies modeled radiation exposures to define internal dose and algorithms were developed so that the readout from portable detector could be translated into the dose delivered to internal target organs.

It is the internal dose (the concentration of the agent at the cell/organ/tissue being affected) and not the environmental exposure level that is more closely related to toxicological effects. Health-based standard setting and development of health effect reference values are moving to apply emerging advances in exposure and toxicological sciences aimed at a more seamless integration of exposure and internal dose metrics, together with the incorporation, coherent alignment, and coordination of novel data streams [70–72]. Elucidation of the linkages between exposures and adverse effects in humans and the ecosystem will result in a better understanding on which to develop effective management strategies.

**Figure 1:**
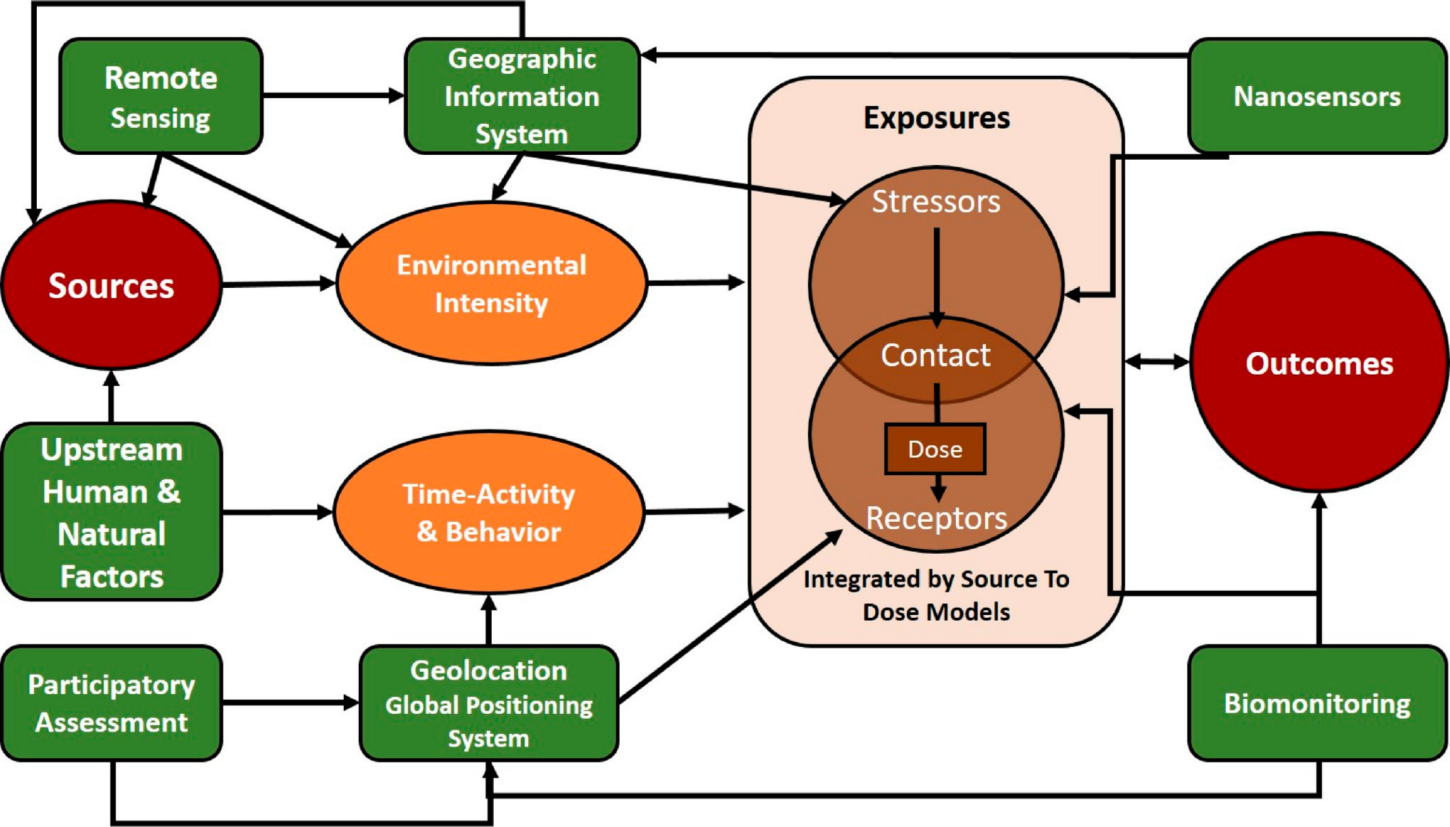
Conceptual integration of exposure and dose (from National Research Council).

Constructing a robust context for this integration calls for coordinated research with human-health and ecologic-health scientists to identify, collect, and evaluate data that capture internal and external markers of exposure in a format that improves the analysis and modeling of exposure–response relationships and links to emerging methods for hazard identification such as high-throughput test systems (HTS). Figure 1 depicts selected scientific and technologic advances for measuring and monitoring considered in relation to a conceptual integration of exposure and dose as espoused by the National Research Council [71].

Mattingly et al. [73] developed an exposure ontology (ExO) designed to address the lack of exposure information required to elucidate environmental contributions to diseases, translate molecular insights from new technologies such as HTS, and aid assessment of human and ecological risks. The ExO formalized definition such as “exposure receptor” and centralized the role of exposure science with the intent to extend its ability to integrate and analyze exposure information within the broader context of environmental health. The exposure receptor can be an organ, tissue or cell, and the exposure stressor can be a biological, physical, or psychosocial agent, so that exposure assessment may include estimating the magnitude, frequency, and duration of an exposure, along with characteristics of the specific receptor. This concept of the exposome is consistent with the life-cycle approach articulated above regarding sensors. Such integration will expand the impact of exposure data and inform existing environmental health data by providing associated real-world exposure context.

On the response side of the exposure-dose-response linkage, adverse outcome pathways (AOP) are emerging as an important construct for the integration of toxicological effects across various levels of biological organization (e.g., genomic, cellular, target tissue, individual, population) based on HTS and identification of molecular initiating events [74,75]. This construct is also completely consistent with the ExO, and more recently Teeguarden et al. [76] have proposed formal linkage of exposure science with the AOP by means of the aggregate exposure pathway (AEP).

Therefore, emerging technologies now provide a method whereby measures of environmental exposure can be translated to an internal dose which can then be used in applications such as biomonitoring of populations or in cell systems. These approaches essentially provide a scalable platform with which to depict both exposure and internal dose. Alignment of exposures across experimental toxicity-testing systems can be achieved by understanding, measuring, and applying this information on the processes that control the time course of concentrations and delivery of chemicals and particles to target cells in various test systems at different scales (e.g., scale of ecological epidemiological studies, target tissue dose for in vivo animal studies, delivered concentration in HTS) that may be used as the basis for developing health-based values to which sensor data may be compared. Such exposure and internal dosimetry considerations can provide a context for the interpretation of emerging sensor data and may also inform future considerations of the form of various health-based reference values.

#### 4.1.1. Dosimeters in the Workplace

The Army projects aimed at developing a naphthalene dosimeter are an example of how biomarkers of exposure are being integrated with chemical sensor-derived data [77]. The first in the series of naphthalene dosimeter projects was funded through the Army’s SBIR program. It developed an instrument capable of measuring naphthalene from air every three minutes [78]. A second project, underway at NIOSH, is an independent evaluation of the performance of the instrument. A third project lead by the U.S. Army Research Institute of Environmental Medicine is deploying the prototype instrument on military fuel-handlers to measure the concentration of naphthalene in their breathing zone. Concurrent to collecting the personal real-time exposure data, biomarkers of naphthalene exposure are being collected from the exhaled breath, urine and skin of the individual wearing the air sensor. Following the radiation dosimetry paradigm, the concurrently collected exposure and biomarker data will be used in a fourth project that will inform development of a model to estimate internal dose [79].

#### 4.1.2. Dosimetry for NAAQS Air Pollutants

Portable sensors available for some NAAQS pollutants (namely, PM, O_3_, and NO_2_) have been evaluated by EPA. There are not specific metabolites that can be used to quantify the general relationship between exposure and dose. However, unlike naphthalene which enters the body by both dermal absorption and inhalation, at least these three NAAQS pollutants enter the body predominately by inhalation. In general, greater than 80% of inhaled ozone is absorbed in the respiratory tract (see Table 5-1 of U.S. EPA, 2013 [80]). Thus, dose can be approximated as the product of O_3_ concentration, minute ventilation, and duration of exposure. Conceivably, an estimate of minute ventilation could be derived using an accelerometer in combination with data on the mass and gender of an individual. A more exact linkage to predicted decrements in lung function due to O_3_ exposure, at least in healthy individuals, could be calculated by linking dosimeter data with an existing model [81]. This type of approach could be applied to both occupational and ambient ozone exposures or adapted for other compounds where health endpoints are closely related to inhaled dose.

Development of a dosimeter for PM is complicated due to the dependence of particle deposition on inhaled particle size, route of breathing, tidal volume, breathing rate, and lung size [82]. A couple of PM samplers that mimic the deposition in adults during nasal breathing and light exercise based on the International Commission on Radiological Protection (ICRP) model [83] have been developed. First, Koehler et al. [84] employed the use of a foam plug in which particle deposition efficiencies are similar to the ICRP [83] predicted total respiratory tract deposition (average of adult males and females) for particles between 0.05 and 2 µm. Total deposited dose in the respiratory tract can be determined either gravimetrically or by digesting the foam and extracting metals or organs for quantification by other means. Second, TSI has developed a Nanoparticle Surface Area Monitor (Model 3550) which can be used to determine particle surface area depositing in the tracheobronchial and alveolar regions of the lung for particles between 0.01 and 1 µm. The Model 3550 is designed to match predicted deposition in an adult male. Its operation is based on the diffusion charging of particles followed by detection of the aerosol using an electrometer. Particle doses can be assessed by the second, computed as a time-weighted average, or cumulative total deposed particle surface area. Available portable PM sensors typically use light scattering to estimate PM mass or number concentration in air for micron-sized or larger particle fractions such as PM_2.5_. Unless an underlying PM size distribution is known or assumed, estimates of dose cannot be derived based on data from these portable PM sensors.

The utility of a dosimeter for ambient NO_2_ is questionable. For NO_2_, one of the critical endpoints is asthma exacerbation via an increase in airways responsiveness, as discussed in Section 5.2.2 of the Integrated Science Assessment (ISA) for NO_2_ [85]. Since the increase in airway responsiveness does not appear to be associated with NO2 dose for NO_2_ concentrations between 100 and 600 ppb [86], it may be sufficient to monitor NO_2_ concentration. Additionally, ambient NO_2_ concentrations are generally only elevated near roadways, as discussed in Section 2.5.3 of the 2016 ISA [85]. An elevated NO2 sensor reading could serve as a warning for a person with asthma to take actions to reduce exposure, e.g., by using recirculation of air in an automobile or avoiding outdoor activities in close proximity to major roadways or during periods of increased traffic.

#### 4.1.3. Dosimeters for Hazardous Air Pollutants (HAPs)

There are 187 air pollutants designated as HAPs, including several VOC, that are emitted by point sources and under the purview of Section 112 of the Clean Air Act [87]. These pollutants are known or suspected to cause cancer or other serious health effects, such as reproductive effects or birth defects, or adverse environmental effects and are often also encountered in the workplace. As sensor technologies emerge to address these pollutants, our recommendations for life-cycle characterization and dosimetry as discussed above will be essential to create proper context for interpretation of sample measurements with comparisons to appropriate health effect reference values, when such values are available [3].

### 4.2. Adopting a Life-Cycle Approach

**Figure 2:**
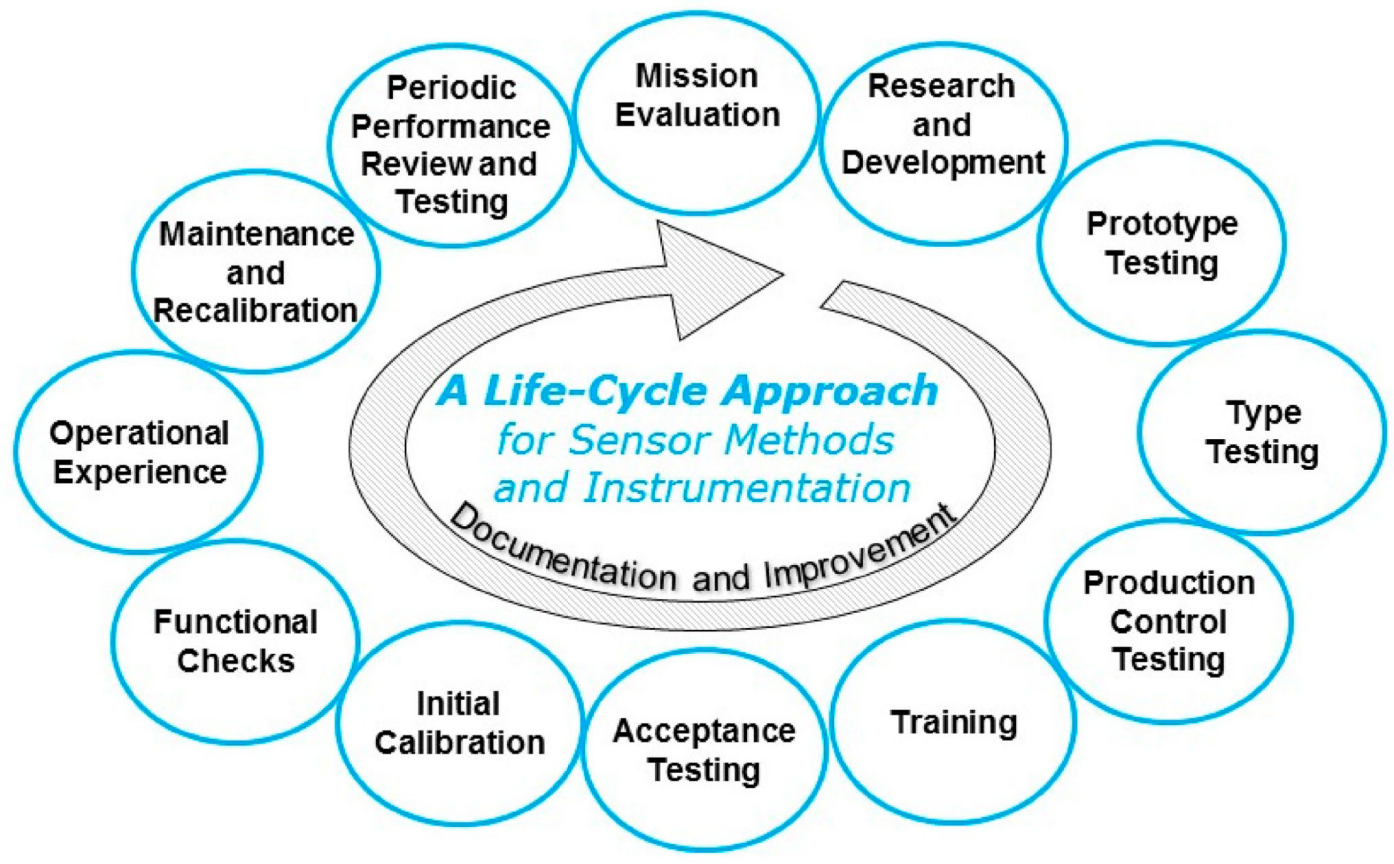
The life-cycle approach for sensor methods and instrumentation [88–91].

An overarching life-cycle framework and decision-making process that the ASHG has encouraged as an ideal for air quality sensor applications is illustrated in Figure 2. The life-cycle concept was originally developed for use in emergency response situations [88], adapted and applied for radioactive air sampling and instrumentation [89], adopted as a systematic way to organize the framework of the White House’s signature initiative on Nanotechnology for Sensors and Sensors for Nanotechnology [90], and most recently expanded to meet all manner of the emerging sensor needs for safety, health, well-being and productivity [91]. The lifecycle begins with a clear and complete identification of the purpose of the measurement, including what needs to be measured, under which conditions it needs to be measured, and how well it needs to be measured. The lifecycle guides the research and development, prototype testing, qualification type testing, production control testing, and training needs for the sensor system. The lifecycle further defines procedures for acceptance testing, initial calibration, functional checks, conduct and evaluation of operational experience, maintenance and recalibration, and periodic performance testing to confirm continued successful use of the sensor system. Effective following of the life-cycle process ensures that the sensor methods and instrumentation will work as intended under realistic conditions. Documentation and continuous improvement are essential at each step.

Use of the lifecycle supports an approach to sensor methods and instrumentation that is consistent with the roles served by resources such as *My Air, My Health: An HHS/EPA Challenge* [92] to develop and validate new methods, and the online *AirNow* [93] maps and forecast data that are collected and shared using federal reference or equivalent monitoring techniques or techniques approved by the state, local or tribal monitoring agencies.

#### 4.2.1. A Working Definition of Air Quality Sensors and Health Informatics

A proposed working definition of *Air Quality Sensors and Health Informatics* might be “the science and practice of determining which information is relevant to meeting air sensor and health objectives; developing and implementing effective mechanisms for collecting, validating, storing, sharing, analyzing, modeling, and applying the information; and then confirming that appropriate decisions were made and that desired mission outcomes were achieved” [94]. The additional steps in the informatics lifecycle include “conveying experience to the broader community, contributing to generalized knowledge, and updating standards and training” [95]. Successful informatics endeavors will apply all of the steps in the process. ASHG members have also assisted with the development of an attempt to define “data readiness levels”, which could help with relevance and reliability issues for the collection, sharing, and application of air sensor data [96].

#### 4.2.2. Roles and Responsibilities of Sensor and Data Customers, Creators, Curators, and Analysts

**Figure 3:**
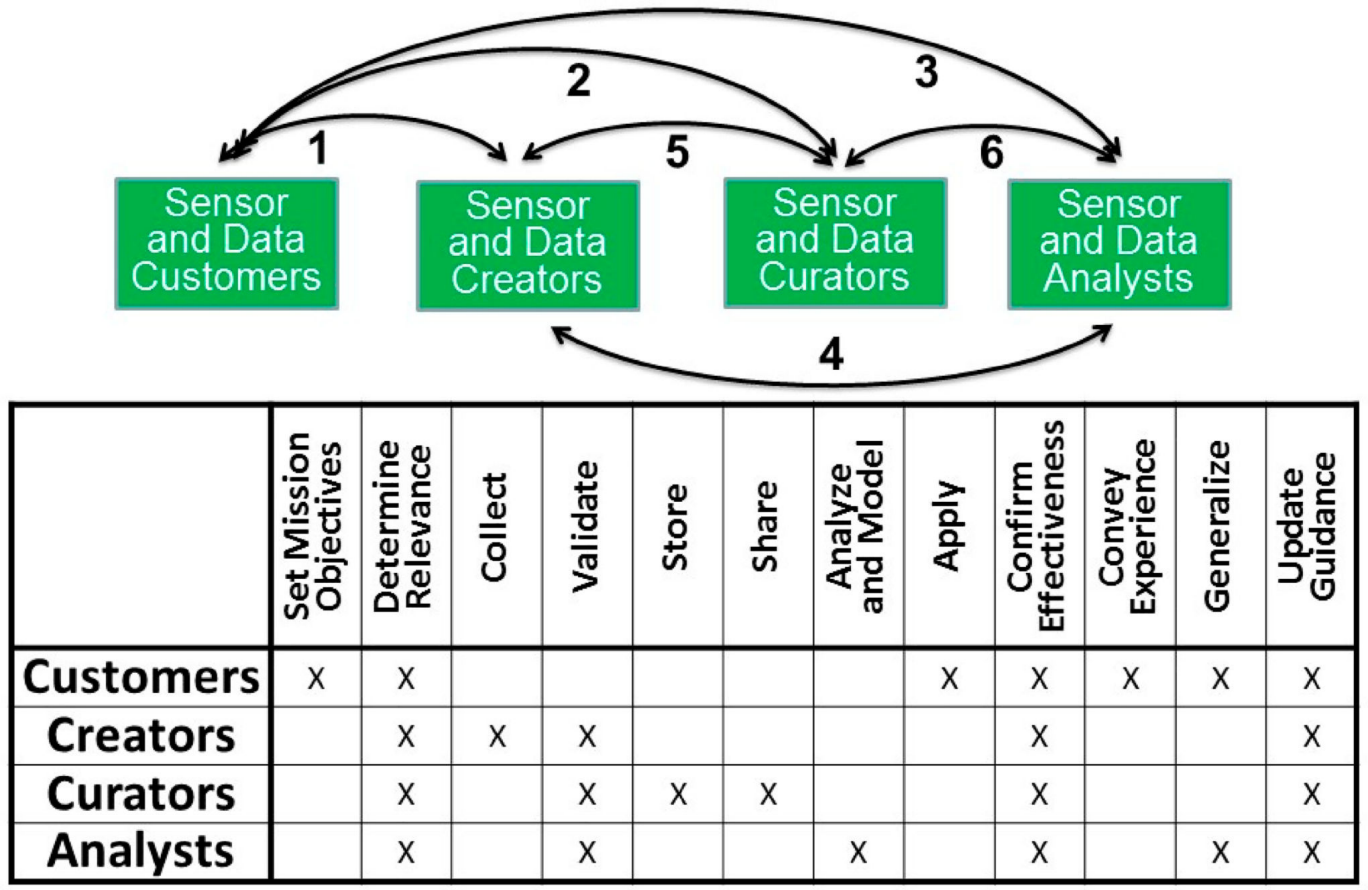
Communication interfaces, roles, and responsibilities for air quality sensor and health data customers, creators, curators, and analysts [95].

In the context of our working definition of informatics for air sensors and health, the roles and responsibilities of the myriad individuals who are engaged in the development and application of air quality sensors can be viewed as fitting into four categories: sensor and data customers (who specify the sensors and data needs for their intended purposes), sensor and data creators (who will develop relevant and reliable sensors and data to meet the customer needs), sensor and data curators (who will maintain and ensure the quality of the sensors and sensor data), and sensor data analysts (who will develop and apply models for data analysis and interpretation that are consistent with the quality and quantity of the data and that those data meet the customers’ needs). In some instances, the same individuals may perform all roles, and in the larger global reality the individuals and their roles may extend over significant distances, organizations, and time periods. As shown in Figure 3, effective communication across the many customer, creator, curator, and analyst interfaces is essential, and that communication across each of the six interfaces must work effectively in both directions [95]. This vision follows the views of Hendren et al. [97] on a collaborative approach to assessing, evaluating, and advancing the state of the field for data curation in the emerging field of nanomaterials and nanotechnology.

## 5. Forecast of Advancing Technologies

Major areas of opportunity to advance the state of the art and application of sensor technologies include: strengthening and sustaining infrastructures for investments and collaborations, improving the sharing of information, integrating data and a coherent interpretation across various venues to construct a cumulative accounting of exposures, and advancing breakthroughs in miniaturization of sensor systems. At this writing, there are efforts across multiple organizations (public and private; large and small; formal and informal) with additional involvement by independent inventors. Entrepreneurial organizations such as Aclima have become involved in collecting data from mobile sensors attached to Google street-view cars [98] and are leading the way to making those data accessible.

Equally important are opportunities to increase the linkage of measurements of exposure via sensor readings to additional parameters useful to estimate dosimetry: biometric data (e.g., pulse rate, breath rate, and the like, which are now available from devices such as FitBit); geospatial measurements of location and daily movement patterns; and of the physical environment (temperature, humidity, etc.). These capabilities are most actively being developed within the occupational exposure arena but migration of these technologies to the low-cost sensor market is anticipated as value-added features. Properly informing and providing useful guidance to ensure these technologies are appropriately engaged will be another challenge for groups like the ASHG.

### 5.1. Infrastructure Needs

#### 5.1.1. Forecast and Statement of Needed Investment

When real-time sensing is combined with concurrent GPS coordinates, the derived data are multidimensional. Today, sensor-derived data include streaming of latitude, longitude, altitude in addition to the chemical data. However, the hardware and software to fully capture the potential usefulness is lacking. For example, visualization of multidimensional data might be imaged as a video of a multi-colored topographical map that flexes as an individual moves through different levels of the positional and chemical data. Depth of color might represent various concentrations of chemicals detected by the sensors. If the full potential is to be captured, investment is essential. Infrastructure is needed to not only capture multidimensional data streams but also analyze it as it is being produced. This is especially relevant to homeland security and for rapid response following a catastrophic event. The application of sensor technology to the field of epidemiology can also be cited as an investment opportunity. New evidence of cause-and-effect will become evident by developing the ability to retrieve archived multidimensional sensor-derived data and overlaying it with geographical disease prevalence information. Challenges of scalability across dimensions of time and geography must be addressed to properly interface with available health-based standards and reference values.

As described in Section 3.1, there is great interest, and investment, in transforming toxicity testing with the application of high throughput analyses using cell cultures [70]. Generating evidence to demonstrate the validity of these testing methods is an ongoing challenge. In this context, we have an opportunity to transform systematic hazard identification and risk assessment processes by combining real-time exposure monitoring with real-time image analysis. Again, investment in the data capture, analytics and archiving capabilities is essential if this opportunity is to be realized.

#### 5.1.2. Scientific Literature Collection and Coordination

Another essential investment is needed to network the fields of Health Science, Electrical Engineering and Computer Science. A first step might be to encourage sensor-related journals to petition the National Library of Medicine for incorporation into the database. The National Library of Medicine’s PubMed database [99] provides the ability to search for peer-reviewed publications. This includes research about the use of sensors to assess indoor and outdoor air. As an example, PubMed includes air sensor-related publications from a journal called “Environmental Monitoring and Assessment” such as this 2017 one about “Public engagement on urban air pollution: an exploratory study of two interventions” [100]. However, not all scientific journals are indexed in PubMed and this is especially true for journals specializing in emerging areas and technologies Editors and publishers can submit their journals for inclusion in PubMed; however, not all journals will be accepted based on NLM’s selection criteria [101]. The relatively new PubMed Commons allows for the sharing of opinions and information about PubMed citations [102]. This could include comments about the techniques used in a publication or alerting readers to consider looking at publications with more current approaches and findings.

### 5.2. Miniaturization

Miniaturization has been essential to recent successes in moving laboratory-scale technologies to the field. For example, to meet the demands for personal exposure monitoring of the size characteristics and spatial distribution of ultrafine particles, Fierz et al. [103] have developed a compact, real-time instrument called the diffusion size classifier (DiSC) which provides particle size information that is in good agreement with the much larger and more expensive laboratory-based aerosol spectrometers. Another personal monitoring device recently released into the market is the portable aerosol mobility spectrometer (PAMS) which simultaneously measures the number-weighted size distribution of submicrometer aerosol, including the nanoparticle fraction [104]. In developing the PAMS, NIOSH investigators used miniaturization to overcome the prohibitive size, weight, and cost limitations of the previous technology, and they also eliminated burdensome regulatory and administrative limitations for record-keeping and transportation of the instrument by replacing the traditionally used radioactive source for particle charge conditioning with a nonradioactive bipolar diffusion charger. Compared to traditionally used instruments, these innovations provided reductions in size (by a factor of 20), weight (by a factor of 10–15), and cost (by a factor of 4), along with improvements in analytical performance. However, further improvements in instrument size, weight, cost, and performance are still needed to make these technologies more widely affordable, deployable, and able to operate in the wide range of particle number concentrations that can be encountered in both workplaces and ambient environments. Needed improvements include smaller, more reliable power sources, including the possibility of body-heat or motion-driven power sources, and more compact data collection, processing, and memory capabilities. As described in the perspective article by Fadel et al. [105], advances in nanotechnologies could enable and accelerate the development of inexpensive, portable devices for the broad detection, identification, and quantification of biological and chemical substances, including sensors for air quality and health applications.

## 6. Summary and Conclusions

The goals of the ASHG are to provide an open dialogue from the multiple disciplines and agencies represented on a number of related issues: (1) improving the understanding of what sensor technologies may be able to deliver for these agencies to meet their missions; (2) communication of what small-scale sensor readings can provide to a number of audiences; and most centrally, (3) best practices in communicating health-related messages to numerous audiences.

In this paper, we have attempted to cross-reference other related projects and provide additional resources to an interested reader to pursue additional information from credible sources. Additional aims were to provide an update on the advances made to date under the auspices of these various programs, to forecast potential applications of rapidly emerging sensor technologies and to foster a collaborative response to challenges involved in their application to research and support of decisions related to air quality management.

## Appendix A

Standards for equipment used to detect and determine toxic gases and vapors in a workplace or similar situation.

### A.1. Published

Europe:

1. EN 45544:2000 Workplace atmospheres. Electrical apparatus used for the direct detection and direct concentration measurement of toxic gases and vapours. Part 1: General requirements and test methods; Part 2: Performance requirements for apparatus used for measuring concentrations in the region of limit values; Part 3: Performance requirements for apparatus used for measuring concentrations well above limit values; Part 4: Guide for selection, installation, use and maintenance.

USA

1. Underwriters Laboratory: UL 2075 Gas and Vapor Detectors and Sensors
2. American National Standards Institute/International Safety Association: ANSI/ISA-92.00.01-2010 Performance Requirements for Toxic Gas Detectors; ANSI/ISA 92.00.02-2013 Installation, Operation, and Maintenance of Toxic Gas-Detection Instruments
3. American Society for Testing and Materials: ASTM E2885-13 Standard Specification for Handheld Point Chemical Vapor Detectors (HPCVD) for Homeland Security Application

International Electrotechnical Commission:

1. IEC 60079-29-1:2007 Explosive atmospheres—Part 29-1: Gas detectors—Performance requirements of detectors for flammable gases
2. IEC 60079-29-2:2007 Explosive atmospheres—Part 29-2: Gas detectors—Selection, installation, use and maintenance of detectors for flammable gases and oxygen

Other:

1. Australian/New Zealand Standard: AS/NZS 4641:2007 Electrical apparatus for the detection of oxygen and other gases and vapours at toxic levels—General requirements and test methods.
2. ISO database: https://www.iso.org/committee/52702/x/catalogue/p/0/u/1/w/0/d/0
3. CEN Database: https://standards.cen.eu/dyn/www/f?p=204:7:0::::FSP_ORG_ID:6245&cs=178094E67E1897102F190938A48C7A285

### A.2. Standards Proceeding through Process

ISO/IEC (IEC 62990-1) Workplace Atmospheres—Part 1: Gas detectors—Performance requirements of detectors for toxic gases

ISO/IEC (IEC 62990-2) Work-place Atmospheres—Part 2: Gas detectors—Selection, installation, use and maintenance of detectors for toxic gases and vapours and oxygen sensors.
